# High-performance RT-DETR model for industrial defect detection with semantic guidance and hierarchical attention

**DOI:** 10.1371/journal.pone.0348807

**Published:** 2026-05-12

**Authors:** Jin Huang, Bin Gu, Yunlong Tang, Bingyue Xu, Puxuan Li, Jiayu Ye, Jingwen Xu, Taihua Zhang

**Affiliations:** School of Electronics and IoT Engineering; ChongQing Industry Polytechnic University,‌‌ Chongqing, ‌‌China; Beijing University of Posts and Telecommunications, CHINA

## Abstract

With the advancement of intelligent manufacturing and Industry 4.0, surface defect detection plays a critical role in ensuring product quality and production safety. To address the limitations of existing detection models in handling small sample sizes, complex textures, and multi-scale defects, this paper proposes a high-performance industrial defect detection model based on the RT-DETR framework, incorporating semantic guidance and hierarchical attention mechanisms. Specifically, a Semantic-Guided Query Enhancement Module is designed to strengthen the contextual awareness of query vectors through multi-source semantic paths and a residual feedback structure. Additionally, a Hierarchical Attention Fusion Structure is constructed to build interactive graphs among multi-scale features, achieving cross-scale semantic alignment and structural consistency modeling. Experiments conducted on three benchmark industrial defect datasets—NEU-DET, DAGM2007, and PCB-DET—demonstrate the effectiveness of the proposed method, achieving mAP@0.5 scores of 78.9%, 84.7%, and 87.4%, respectively, outperforming the best baseline models by 1.2% to 3.0%. For the more stringent mAP@0.5:0.95 metric, the method achieves 44.3%, 48.1%, and 52.3%, significantly surpassing mainstream models such as YOLOv8, and BMA-YOLO. Furthermore, Grad-CAM visualizations validate the model’s superior focus capability and boundary-fitting accuracy in regions with complex textures and sparse targets. Overall, the proposed architecture enhances semantic perception, scale robustness, and generalization performance in industrial defect detection while maintaining real-time efficiency.

## 1. Introduction

In recent years, with the rapid development of intelligent manufacturing and Industry 4.0, surface defect detection has become a critical step in ensuring product quality and production safety. It also serves as a key technical support for building flexible production lines and enabling autonomous equipment diagnostics [1]. However, industrial defects often exhibit irregular shapes, extreme scale variations, and complex background textures. Most traditional inspection systems still rely on manual rechecking, which fails to meet the practical demands of high speed, high precision, and low cost [2]. Therefore, developing defect detection algorithms with real-time performance, fine-grained capability, and portability is of great significance for improving quality control and predictive maintenance in manufacturing.

Although deep learning methods have shown strong performance in general object detection tasks, directly applying them to industrial surface defect scenarios still faces three major challenges. First, backbone networks based solely on convolution or pure Transformers often struggle to balance local textures and global semantics. This leads to unstable performance when detecting both tiny cracks and large-scale defects simultaneously [3]. Second, existing multi-scale fusion strategies easily introduce redundant information, and the localization becomes less accurate when defects appear elongated or aligned with texture directions [4]. Third, industrial datasets suffer from noisy labels and imbalanced distributions. Accelerating convergence and improving generalization remain unresolved challenges [5]. Furthermore, traditional digital signal processing methods for defect detection are often limited in adaptability and accuracy under complex conditions [6], while earlier surveys highlight the long-standing challenges in automating visual inspection [7].

To address the above issues, this paper proposes an improved model for industrial defect detection based on the RT-DETR framework. While maintaining real-time performance, the model introduces a query enhancement mechanism guided by external semantic priors and a hierarchical attention fusion structure. These modules effectively improve the recognition and localization accuracy of heterogeneous-scale defects. The model can be flexibly integrated into existing pipelines without additional computational overhead and demonstrates strong robustness on small-sample and imbalanced data.

- **We propose a Query Enhancement Mechanism with Semantic Guidance**, which injects prior semantic vectors to dynamically reconstruct detection queries. This enhances the model’s receptive field and contextual representation for hard-to-detect details.
- **We design a Hierarchical Attention Fusion Structure to Enhance Multi-Scale Defect Modeling Capability**, which builds cross-scale attention interactions between shallow textures and deep semantics. It guides the network to maintain high-confidence predictions for both tiny cracks and large-area peeling defects.
- **We construct a unified inference framework and validate it on NEU-DET, DAGM2007, and PCB-DET datasets**. Experimental results show that our method significantly outperforms mainstream algorithms on both mAP@0.5 and mAP@0.5:0.95 metrics, demonstrating its generality and efficiency in complex industrial scenarios.

## 2. Related work

### 2.1. Research progress in object detection algorithms

#### 2.1.1. Two-stage object detection algorithm.

In recent years, two-stage object detection algorithms have received continued attention from both academia and industry due to their superior performance in accuracy and localization. These methods typically involve two phases: proposal generation and object classification. They demonstrate strong feature representation capabilities and precise localization, making them widely used in complex detection tasks. Zhou et al. [8] proposed the Probabilistic Two-Stage framework, which introduces a probabilistic modeling mechanism during proposal generation. This approach significantly improves the detector’s ability to handle objects with blurred boundaries. Duan et al. [9] designed the Corner Proposal Network for anchor-free two-stage detection. By using a direction-sensitive box regression strategy, it improves the accuracy of rotated object detection. Yu et al. [10] proposed a two-stage approach tailored for small-object detection tasks. It integrates multi-scale contextual information to enhance proposal features and address the challenges of small object recognition.

To meet the demands of detection in complex environments, several studies have explored adaptive optimization and generalization of two-stage structures. Ouyang [11] combined the efficiency of YOLO with the DETR framework and introduced DEYO. This model performs localization and classification tasks in a step-wise decoupled manner. It balances detection speed and structural interpretability. Al-Refai et al. [12] focused on detection under low-light conditions. They constructed a deep network that integrates image enhancement and two-stage detection. This improves the model’s ability to detect weak texture targets. Sun et al. [13] analyzed the evolution of object detection frameworks, and highlighted structural optimization directions. Zhang et al. [14] proposed PNANet by integrating pyramid non-local attention. It enhances cross-layer semantic fusion while maintaining accuracy.

In addition, several review and comparative studies have provided systematic summaries of the development trends of two-stage detection models. Carranza-García et al. [15] evaluated the performance of two-stage and one-stage detectors in autonomous driving. They analyzed detection accuracy, inference speed, and resource usage. Han [16] conducted a comparative evaluation of two-stage and one-stage detection models. The analysis covers detection accuracy, inference speed, and resource consumption to explore their application differences. Lu et al. [17] proposed MimicDet, bridging the gap between one-stage and two-stage paradigms by distilling two-stage representations. In summary, two-stage detection algorithms are evolving toward lightweight design, scene adaptability, and modular collaboration. They provide critical support and technical reserves for industrial-grade detection systems.

#### 2.1.2. One-stage object detection algorithm.

In recent years, one-stage object detection algorithms have gained significant attention due to their simple end-to-end structure and fast inference speed. These algorithms directly perform bounding box prediction and category classification from raw images through a unified network architecture. They offer strong real-time performance and deployment flexibility in industrial applications. Dai et al. [18] proposed the Dynamic Head structure, which integrates multiple attention mechanisms into the detection head to achieve unified task modeling. It improves multi-scale object recognition while maintaining inference speed. Feng et al. [19] designed the TOOD framework to address task misalignment by using a task-adaptive feature assignment strategy. This reduces localization errors. Hu et al. [20] proposed AFDetV2 for point cloud detection. Their study showed that the second stage can be removed in some cases without performance degradation, expanding the applicability of one-stage frameworks.

To meet the requirements of complex industrial backgrounds and real-time performance, the YOLO series has undergone continuous upgrades. Li et al. [21] introduced a more efficient backbone and lightweight decoder in YOLOv6. This significantly improved recognition efficiency and stability in industrial defect detection. Wang et al. [22] proposed YOLOv7 with a trainable Bag-of-Freebies strategy. It balances speed and accuracy and has become a representative method for real-time detection. More recently, Wang et al. [23] proposed YOLOv10 as a real-time end-to-end detection framework, further streamlining training and inference while maintaining competitive accuracy. Ali and Zhang [24] reviewed lightweight detection models and pointed out that one-stage detectors have broad application prospects in resource-constrained environments such as mobile and edge devices. These models are expected to play a key role in intelligent terminal object recognition. In addition, Jegham et al. [25] systematically reviewed the evolution from YOLOv1 to YOLOv12. Their work emphasized that network architecture optimization and feature assignment strategies are crucial to improving detection accuracy while maintaining efficiency.

Beyond specific models, several studies have discussed the development trends and challenges of one-stage detection from a broader perspective. Shi et al. [26] examined the performance of one-stage frameworks in small-object detection scenarios and proposed an efficient solution. Oksuz et al. [27] reviewed imbalance problems in object detection and analyzed strategies such as focal loss and resampling to improve robustness. Edozie et al. [28] emphasized potential bottlenecks in data dependency and class imbalance. They suggested the adoption of domain adaptation and weak supervision to improve generalization. In industrial defect inspection, transformer-based one-stage designs have also been explored; for example, Huang et al. [29] introduced an adaptive cross transformer with contrastive learning to enhance feature discrimination under complex textures and subtle defect patterns. In summary, one-stage object detection algorithms are evolving toward better structural optimization and semantic modeling. They aim to achieve higher accuracy and broader adaptability while maintaining efficiency.

### 2.2. Industrial surface defect detection

In recent years, industrial surface defect detection has become a research focus in the field of computer vision, as it plays a vital role in ensuring product quality in intelligent manufacturing. Deep learning methods, with their powerful capabilities in feature extraction and pattern recognition, have been widely applied to defect detection tasks involving various industrial materials such as metal, plastic, PCB, and fabric. Božič et al. [30] proposed a mixed supervision architecture to address the challenge of detecting small-scale surface defects with limited annotations. Martin et al. [31] outlined deep learning strategies tailored for industrial surface defect detection systems. Xu et al. [32] proposed a self-supervised surface defect detection approach using a segmentation network, enabling effective training without manual annotations. To further enhance representation robustness under complex textures, Zhou et al. [33] introduced a transformer framework that combines global dual attention with local representations, improving defect localization and discrimination.

To address the difficulty of obtaining labeled data in real-world industrial scenarios, some studies have explored self-supervised and few-shot learning mechanisms to reduce the dependence on annotations. Jin and Chen [34] conducted a survey of surface defect detection techniques using a small number of labeled samples. Sun et al. [35] introduced a knowledge distillation framework for incremental few-shot surface defect detection, improving performance under limited data conditions. Wang et al. [36] proposed a fine-tuned few-shot network for steel defect detection, integrating scale attention modules for improved generalization.

As model architectures continue to evolve, multi-scale feature fusion and frequency-domain modeling have also become key directions for enhancing detection accuracy. Min et al. [37] proposed FS-RSDD, a few-shot detection method for rail surfaces based on prototype learning. Peng et al. [38] presented a multi-scale focusing and enhancement GANomaly architecture for robust localization and classification in complex defect scenes. Along this line, Zhou et al. [39] proposed IFIFusion to integrate independent feature information for more effective defect representation and decision making, while Liu et al. [40] developed a global attention module with a cascade fusion network to improve steel surface defect detection under multi-scale variations. In addition, Huang et al. [41] designed SSA-YOLO as an improved one-stage detector for hot-rolled strip steel, enhancing real-time defect detection performance via attention-enhanced feature learning. In summary, research on industrial surface defect detection is gradually shifting from static image analysis to more diverse directions, including multi-source modeling, multi-scale learning, low-label supervision, and continuous spatiotemporal representation.

## 3. Method

### 3.1. Overall model architecture

**Fig 1:**
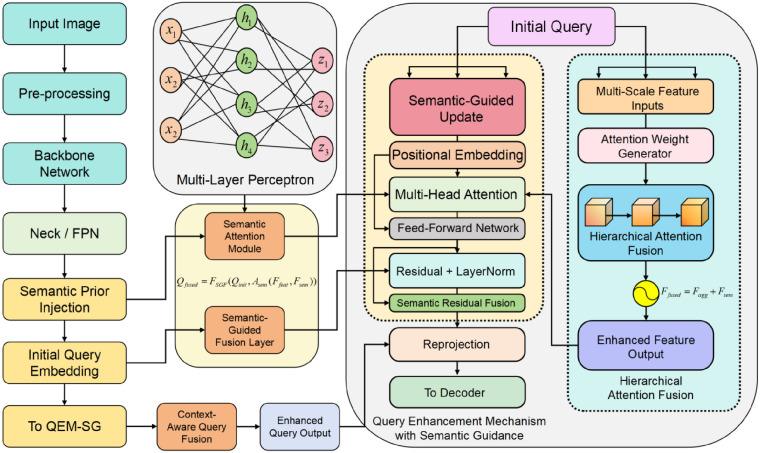
The proposed industrial defect detection architecture integrates semantic guidance and hierarchical attention. It combines a query enhancement mechanism and a multi-scale feature fusion module to improve detection accuracy and structural modeling capability.

To address the dual challenges of fine-grained semantic perception and multi-scale structural fusion in industrial surface defect detection, this paper proposes a high-performance RT-DETR architecture that integrates semantic guidance and hierarchical attention. The proposed method consists of two key modules. First, a Query Enhancement Mechanism with Semantic Guidance (QEM-SG) is designed. It introduces a semantic attention module and a semantic fusion layer to inject semantic priors and guide dynamic features during the encoding stage. This improves the contextual adaptability and discriminative capability of the queries. Second, a Hierarchical Attention Fusion (HAF) structure is constructed. It incorporates an attention weight generator and a residual feature aggregation mechanism to selectively fuse multi-scale features and enhance the model’s ability to capture cross-scale structures. The overall framework emphasizes bidirectional coupling between semantics and structure, along with collaborative optimization across modules. This enables the downstream decoder to access more discriminative and multi-level contextual information when detecting defect regions. The complete architecture is illustrated in Fig 1.

### 3.2. Query enhancement mechanism with semantic guidance

**Fig 2:**
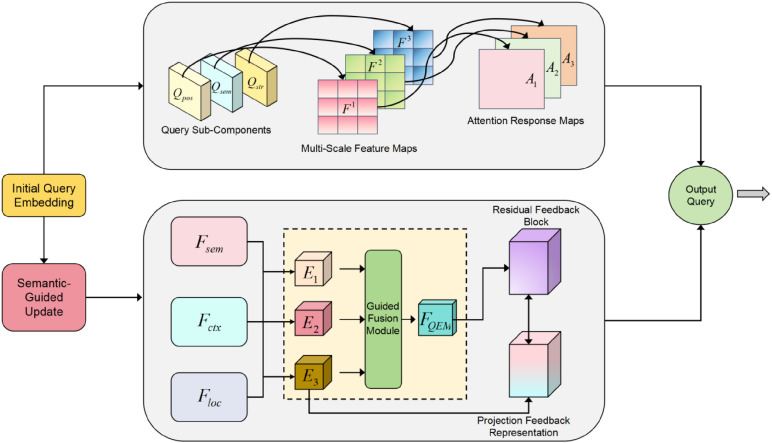
The proposed framework of the query enhancement mechanism with semantic guidance aims to optimize the initial query representation through multi-source semantic fusion and residual feedback. The semantic pathways are implemented by lightweight attention blocks operating on query tokens, and semantic priors are derived from multi-scale encoder features via pooling and linear projection within the same network. This design enhances the modeling capability of multi-scale defect features and improves the consistency of attention responses.

To further enhance the model’s ability in multi-scale feature modeling and semantic representation consistency, this study introduces a Query Enhancement Mechanism with Semantic Guidance into the overall architecture. The model architecture diagram is shown in Fig 2.

Based on the initial query embeddings, this module performs structural decomposition and guided fusion. It jointly models contextual, structural, and semantic information. This enables more discriminative query representations for multi-scale attention. The initial query is first processed by three semantic pathways to extract fine-grained semantic features. These features are then fused to generate projectable enhanced query representations.

Let the initial query embedding be $Q\in {\mathbb{R}}^{N\times d}$. Let the multi-scale encoder outputs be $\{X^{\left(l\right)}{\}}_{l=1}^{L}$, where $X^{\left(l\right)}\in {\mathbb{R}}^{H_{l}W_{l}\times d}$. A global semantic prior token $P_{g}\in {\mathbb{R}}^{1\times d}$ and a regional semantic prior set $P_{r}\in {\mathbb{R}}^{M\times d}$ are derived from $\{X^{\left(l\right)}{\}}_{l=1}^{L}$ by pooling and linear projection as follows:


$$P_{g}=W_{g}\cdot \frac{1}{\sum_{l=1}^{L}H_{l}W_{l}}\sum_{l=1}^{L}\sum_{u=1}^{H_{l}W_{l}}X_{u}^{\left(l\right)}$$ (1)

$$P_{r}(m)=W_{r}\cdot \frac{1}{\sum_{l=1}^{L}|\Omega_{m}^{\left(l\right)}|}\sum_{l=1}^{L}\sum_{u\in \Omega_{m}^{\left(l\right)}}X_{u}^{\left(l\right)},\;m\in \{1,\ldots ,M\},$$ (2)

where $\Omega_{m}^{\left(l\right)}$ denotes the index set of the *m*th coarse region on the *l*th scale feature map, and $W_{g},W_{r}\in {\mathbb{R}}^{d\times d}$ are *l*earnable projections.

First, the initial query representation is input into a semantic-guided update module. This module contains three semantic pathways that extract semantic features *F*_sem_, contextual features *F*_ctx_, and local structural features *F*_loc_. The three types of semantic pathways can be represented in the feature space as follows:


$$F_{sem}=\phi_{sem}(Q)$$ (3)

$$F_{ctx}=\phi_{ctx}(Q)$$ (4)

$$F_{loc}=\phi_{loc}(Q)$$ (5)

The semantic pathway is parameterized by cross attention from query tokens to the semantic priors, followed by a feed forward block:


$${\tilde{Q}}_{sem}=LN(Q+CA(Q,\;K=[P_{g};P_{r}],\;V=[P_{g};P_{r}]))$$ (6)

$$F_{sem}=LN({\tilde{Q}}_{sem}+FFN({\tilde{Q}}_{sem}))$$ (7)

The contextual pathway is parameterized by cross attention from query tokens to a context memory constructed from multi-scale encoder features:


$$C_{ctx}=Concat(Pool(X^{\left(1\right)}),\ldots ,Pool(X^{\left(L\right)}))W_{c},\;C_{ctx}\in {\mathbb{R}}^{T\times d}$$ (8)

$$F_{ctx}=LN(Q+CA(Q,\;K=C_{ctx},\;V=C_{ctx}))$$ (9)

where Pool(·) denotes pooling with a fixed output length, $W_{c}\in {\mathbb{R}}^{d\times d}$ is a learnable projection, and *T* is the number of pooled context tokens.

The local structural pathway is parameterized by windowed self attention on the query sequence with window size *w*, followed by a linear projection:


$$F_{loc}=LN(Q+{WSA}_{w}(Q))W_{l}$$ (10)

where $W_{l}\in {\mathbb{R}}^{d\times d}$ is learnable.

Next, the outputs of the three branches are passed through structure-aware encoders *E*_1_, *E*_2_, *E*_3_ for representation compression and semantic mapping. This yields:


$$Z_{i}=E_{i}(F_{i}),\;i\in \{1,2,3\}$$ (11)

Each $E_{i}(\cdot )$ is implemented as a projection and feed forward block:


$$E_{i}(F)=LN(FW_{i})+FFN(LN(FW_{i})),\;W_{i}\in {\mathbb{R}}^{d\times d}$$ (12)

Then, the projected features are input into a Guided Fusion Module. This module uses semantic-driven attention weighting to generate the fused representation *F*_QEM_:


$$F_{QEM}=𝒢(Z_{1},Z_{2},Z_{3})$$ (13)

The fusion weights are computed by token-level scoring and softmax normalization:


$$\alpha_{i}=\frac{\exp(s_{i})}{\sum_{j=1}^{3}\exp(s_{j})},\;s_{i}=\frac{1}{N}\sum_{n=1}^{N}\;w^{⊤}\tanh\;(WZ_{i}^{\left(n\right)}),\;i\in \{1,2,3\}$$ (14)

$$F_{QEM}=\alpha_{1}Z_{1}+\alpha_{2}Z_{2}+\alpha_{3}Z_{3}$$ (15)

To build a consistent contextual representation for the enhanced query, *F*_QEM_ is fed into a Residual Feedback Block. It is combined with the previous projected representation *F*_proj_ for joint modeling:


$$F_{res}=\Psi (F_{QEM},F_{proj})$$ (16)

The residual feedback is implemented by a gated combiner:


$$G=\sigma \;([F_{QEM};F_{proj}]W_{f}+b_{f}),\;F_{res}=G⊙F_{QEM}+(1-G)⊙F_{proj}$$ (17)

Finally, the residual path outputs the enhanced query representation *Q*_out_, which serves as the input to the next stage of multi-scale attention:


$$Q_{out}=Q+F_{res}$$ (18)

This module is plug-and-play and scalable. It can be integrated into the overall architecture as a semantic complementation mechanism for the initial query embeddings *Q*. To further improve cross-scale fusion, the enhanced query *Q*_out_ is decomposed into three sub-queries:


$$Q_{out}\to \{Q_{pos},Q_{sem},Q_{str}\}$$ (19)

The sub-queries are obtained by linear projections with preserved dimensionality:


$$Q_{pos}=Q_{out}W_{pos},\;Q_{sem}=Q_{out}W_{sem},\;Q_{str}=Q_{out}W_{str},\;W_{pos},W_{sem},W_{str}\in {\mathbb{R}}^{d\times d}$$ (20)

Through this mechanism, the model incorporates multi-dimensional semantic guidance signals during query generation. This provides strong structural priors and semantic alignment for subsequent cross-scale attention. It improves the robustness and discriminability of object modeling.

### 3.3. Hierarchical attention fusion structure to enhance multi-scale defect modeling capability

**Fig 3:**
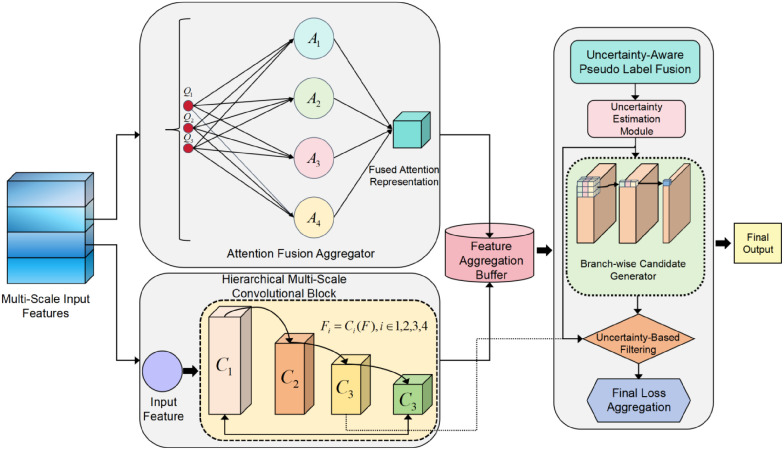
The overall architecture of the proposed Hierarchical Attention Fusion Structure integrates multi-scale convolutional encoding and an attention fusion mechanism. The fused representation is delivered to the Feature Aggregation Buffer for scale aligned consolidation and then mapped by the Branch-wise Generator to form branch specific candidate features. This structure is designed to enhance the representation capability of defect features and to improve semantic consistency modeling.

In multi-scale industrial defect modeling tasks, how to effectively aggregate features from different scales and improve their cross-level semantic consistency remains one of the key challenges in achieving high detection accuracy. To address this issue, we propose a Hierarchical Attention Fusion Structure. The model architecture diagram is shown in Fig 3.

This structure explicitly models attention interactions between multi-scale inputs to build a unified and highly discriminative attention representation space. It is decoupled from the basic convolutional modeling module. It offers good plug-and-play properties and scalability. The structure can be seamlessly integrated into various detection backbones, enabling efficient and generalizable defect representation enhancement.

Specifically, let the input features be a set of multi-scale tensors $\{F_{s}{\}}_{s=1}^{S}$, where each scale corresponds to a semantic representation with a different receptive field level. We first apply projection transformations to obtain the query *Q*_*s*_, key *K*_*s*_, and value *V*_*s*_ representations for each scale:


$$Q_{s}=W_{q}^{\left(s\right)}F_{s}$$ (21)

$$K_{s}=W_{k}^{\left(s\right)}F_{s}$$ (22)

$$V_{s}=W_{v}^{\left(s\right)}F_{s}$$ (23)

Next, we construct a cross-scale attention similarity graph. The response weights between different scale pairs are calculated using scaled dot-product attention:


$$\alpha_{s,t}=Softmax\left(\frac{Q_{s}K_{t}^{⊤}}{\sqrt{d}}\right)$$ (24)

Here, *d* denotes the dimension scaling factor for attention embeddings. To fuse the response features from multiple scales, we introduce a weighted aggregation operation to generate a unified attention fusion representation:


$$A_{s}=\sum_{t=1}^{S}\alpha_{s,t}V_{t}$$ (25)

Subsequently, we use an inter-level aggregator to enhance the interaction between attention representations and generate the final fused feature $\tilde{F}$, which will be used for candidate generation and uncertainty estimation:


$$\tilde{F}=𝒢(A_{1},A_{2},\ldots ,A_{S})$$ (26)

Here, 𝒢(·) denotes a nonlinear composition module. It can be implemented using stacked convolutions or feedforward networks.

To enhance structural stability and semantic alignment of the features, we introduce a residual feedback mechanism. The original input features are injected into the fusion pathway:


$$F_{out}=\tilde{F}+\sum_{s=1}^{S}\gamma_{s}F_{s}$$ (27)

Here, $\gamma_{s}$ are learnable residual modulation coefficients. They dynamically adjust the contribution of each scale to the final fused representation.

Finally, the fused feature is passed to the Feature Aggregation Buffer and Branch-wise Generator modules to enable coupling between upstream and downstream structures and support uncertainty modeling:


$$Z=ℋ(F_{out})$$ (28)

In Fig 3, ℋ(·) corresponds to a two-stage mapping consisting of Feature Aggregation Buffer ℬ(·) and Branch-wise Generator ℛ(·),


$$U=ℬ(F_{out})$$ (29)

Z=ℛ(U) (30)

where ℬ(·) performs scale aligned consolidation by channel mixing and spatial refinement, and ℛ(·) maps *U* into branch specific candidate features required by subsequent candidate generation and uncertainty estimation heads. The Branch-wise Generator can be instantiated as parallel lightweight projections $\{W_{br}^{\left(k\right)}{\}}_{k=1}^{K}$ for *K* branches,


$$Z^{\left(k\right)}=W_{br}^{\left(k\right)}U,\;k\in \{1,\ldots ,K\}$$ (31)

and the overall output is $Z=\{Z^{\left(k\right)}{\}}_{k=1}^{K}$.

Here, ℋ(·) denotes the nested convolutional processing or candidate feature mapping function within the following structures. It corresponds closely to the Feature Aggregation Buffer and Branch-wise Generator in the model diagram.

In summary, the proposed Hierarchical Attention Fusion Structure maintains modular independence while enabling contextual interaction and consistent responses across multi-scale features. It provides a strong semantic foundation for the following uncertainty filtering and label fusion processes.

### 3.4. Training objectives

Based on the previous modules, we construct a unified optimization training framework. This framework aims to enhance feature consistency, improve the discriminability of uncertainty estimation, and increase the robustness of pseudo-label fusion. The training process relies on the fused feature representation 𝐅 generated by the multi-scale convolution module and the semantic-guided attention mechanism. It also incorporates the uncertainty predictions 𝐔 from the multi-branch candidate generator. These predictions guide the pseudo-label filtering and the final supervision process. The goal is to jointly improve the model’s generalization and robustness.

The optimization objective on fully annotated datasets is defined by detection supervision with ground-truth labels and an auxiliary uncertainty consistency regularization across branches.

Let the detection head outputs be denoted by p(𝐅), and let the ground-truth annotations be 𝐘. The supervised detection loss is written as:


$${ℒ}_{det}={ℒ}_{cls}(p(𝐅),𝐘)+{ℒ}_{box}(p(𝐅),𝐘)+{ℒ}_{obj}(p(𝐅),𝐘)$$ (32)

where ${ℒ}_{cls}$, ${ℒ}_{box}$, and ${ℒ}_{obj}$ correspond to the classification, localization, and objectness losses in the improved YOLO paradigm.

To further improve the uncertainty filtering mechanism, we introduce an uncertainty regularization loss ${ℒ}_{\text{unc}}$ to align uncertainty distributions across branches:


$${ℒ}_{\text{unc}}=\frac{1}{K}\sum_{k=1}^{K}\overset{2}{\underset{2}{\left\|{𝐔}_{k}-\bar{𝐔}\right\|}}$$ (33)

Here, *K* is the number of candidate branches. ${𝐔}_{k}$ denotes the uncertainty estimation of the *k*-th branch. $\bar{𝐔}$ is the average uncertainty across branches.

The overall training objective is defined as:


$${ℒ}_{\text{total}}={ℒ}_{det}+\lambda_{u}{ℒ}_{\text{unc}}$$ (34)

Here, $\lambda_{u}$ is the weighting coefficient for the uncertainty regularization term. It controls the relative contribution of ${ℒ}_{\text{unc}}$ to the overall objective. This unified objective function provides a consistent optimization view across modules. It promotes their joint evolution in feature extraction and semantic consistency modeling.

## 4. Dataset and experimental setup

### 4.1. Dataset

#### 4.1.1. NEU-DET.

**Fig 4:**
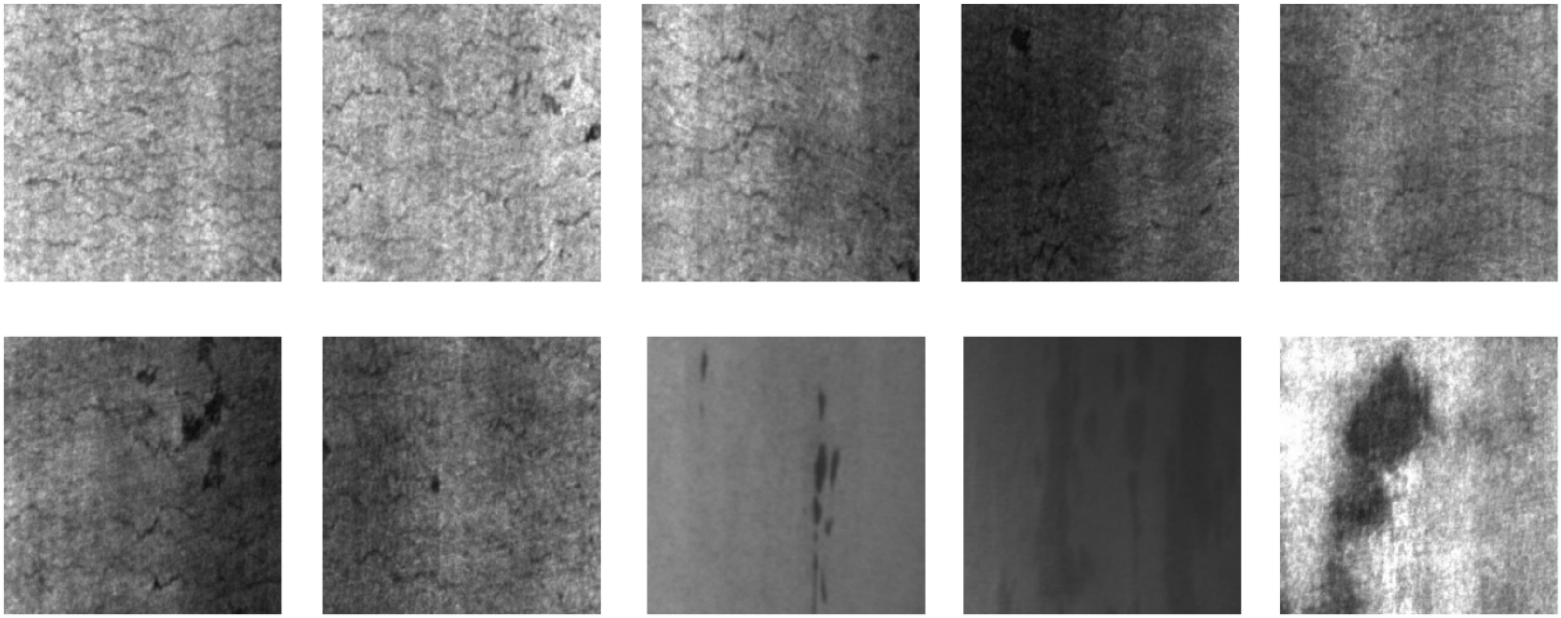
Ten typical defects in NEU-DET.

The NEU-DET dataset [42] provides a high-quality benchmark for typical industrial surface defect detection tasks. It features balanced samples, clear categories, and highly representative defect types. The dataset includes six common categories of steel defects, such as scratches, cracks, and indentations. These defects exhibit significant texture variations and local structural differences. This helps evaluate the capability of detection models in handling fine-grained defects and inter-class interference. Considering that this study focuses on a high-performance industrial defect detection model with semantic guidance and hierarchical attention, the dataset offers a solid testing environment for multi-scale feature modeling and semantic enhancement. It effectively supports model validation on small object recognition and complex background adaptation. The dataset consists of steel images collected from real industrial production lines. The image resolution is fixed at 200×200. Each class contains 300 samples, resulting in a total of 1800 images. The number of samples per class is almost equal, which avoids bias caused by class imbalance during training. Each image contains only a single defect target and has clear category distinction. This provides a stable input foundation for feature extraction and saliency learning. The defect targets in the dataset exhibit diverse sizes, irregular shapes, and complex backgrounds across multiple scales. This makes it suitable for validating the proposed structure in terms of semantic consistency modeling and hierarchical attention fusion.The surface defects of steel in the industrial production process represented by this dataset are shown in Fig 4.

#### 4.1.2. DAGM2007.

The DAGM2007 dataset [43] is widely used for evaluating the performance of high-precision industrial surface defect detection tasks. It contains rich texture variations and complex background noise. This allows for effective testing of detection models in fine-grained defect perception, local structural modeling, and multi-scale region response. The dataset simulates defect recognition under complex backgrounds in real industrial scenarios. It includes various defect types. These range from subtle patterns such as blurred edges and low contrast to strongly distorted regions with significant structural changes. The dataset highlights differences in model capabilities for semantic consistency modeling and hierarchical information fusion. Evaluation based on this dataset enables multi-dimensional validation of the proposed modules in handling cross-scale interference, deformation adaptation, and structural redundancy suppression.

**Fig 5:**
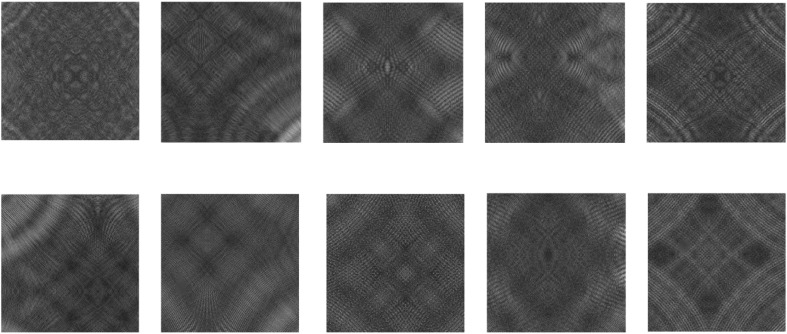
DAGM2007 Ten typical defects of medium and high precision industrial surfaces.

The DAGM2007 dataset contains ten categories. Each category includes 1000 sample images, with 950 being defect-free and 50 containing synthetic defects. The images have a resolution of 512×512. The consistent high resolution ensures the preservation of fine details. The defect types include edge tears, regional wrinkles, and linear cracks. These cover many common industrial defect patterns. To better simulate real-world industrial detection requirements, we divide the training and testing sets based on defect categories. The dataset uses standard object detection annotations for label reconstruction. This setting introduces classification difficulty while also supporting general tasks such as object localization and structural modeling. The dataset provides a strong foundation for validating the performance of multi-scale perception structures.The high-precision industrial surface defects presented in this dataset are shown in Fig 5.

#### 4.1.3. PCB-DET.

**Fig 6:**
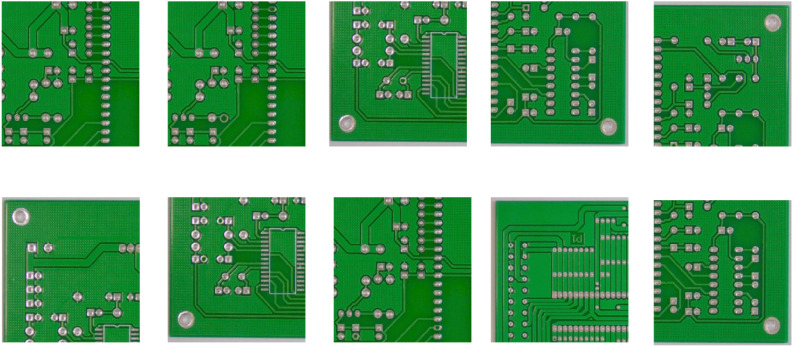
Ten typical defects of printed circuit boards in PCB-DET.

The PCB-DET dataset [44] is built on real industrial images of printed circuit boards (PCBs). It includes various defect categories that are small in size, complex in shape, and sparsely distributed. The dataset exhibits significant structural heterogeneity and class imbalance. These challenges raise the requirements for higher modeling precision and robustness in defect localization and classification. In particular, it serves as a key benchmark for evaluating detection models in terms of multi-scale modeling and fine-grained representation. The dataset provides a rigorous platform to assess the generalization capability of industrial defect detection algorithms. It enables comprehensive validation of algorithm effectiveness under complex textures and non-uniform backgrounds. This dataset contains high-resolution PCB images with annotations of common real-world defect types. These include open circuits, short circuits, missing lines, and extra copper traces. It is suitable for testing the ability of detection models to represent features in fine structural regions. In this study, the PCB-DET dataset is used to evaluate the proposed multi-scale hierarchical attention fusion structure. The focus is on its modeling capability and detection performance in industrial scenarios with strong structural differences, sparse defect regions, and large scale variations.Typical defects in this dataset are shown in Fig 6.

### 4.2. Experimental setup

The proposed defect detection model is implemented and trained using the PyTorch 2.7.0 framework under the Python 3.12 environment on Ubuntu 22.04, with CUDA 12.8 acceleration. All experiments are conducted on a single NVIDIA A100 GPU with 80 GB memory, equipped with 24 vCPU Intel(R) Xeon(R) Platinum 8568Y+ and 120 GB RAM. All input images are resized to 640 × 640 to balance receptive field and computational efficiency. The model is trained for 300 epochs with a batch size of 16 using stochastic gradient descent with momentum, where the initial learning rate is set to 0.01 and dynamically adjusted by a learning rate scheduler to stabilize convergence. During training, multi-scale image input and data augmentation strategies, including random crop, mirror flipping, and color jitter, are applied to improve robustness against defect deformation and appearance variation. In the evaluation phase, Precision, Recall, mAP@0.5, and mAP@0.5:0.95 are adopted to comprehensively assess detection performance under different defect categories and scale conditions. The overall experimental settings are shown in Table 1.

**Table 1:** Experimental Settings of the Proposed Model.

Parameters	Value	Description
Operating System	Ubuntu 22.04	Experimental runtime environment
Python Version	3.12	Programming language environment
Framework	PyTorch 2.7.0	Deep learning implementation framework
CUDA Version	12.8	GPU acceleration toolkit
GPU	NVIDIA A100 80GB × 1	Graphics processing unit used for training and inference
CPU	24 vCPU Intel(R) Xeon(R) Platinum 8568Y+	Central processing unit configuration
Memory	120 GB	System RAM capacity
Initial Learning Rate	0.01	Starting value for SGD optimizer
Optimizer	SGD	Stochastic Gradient Descent
Momentum	0.937	Momentum factor for stable updates
Weight Decay	5 × 10^−4^	Regularization factor
Image Size	640 × 640	Resized input dimension
Batch Size	16	Number of samples per batch
Epochs	300	Total training epochs
Data Augmentation	Random Crop, Flip, Color Jitter	Enhance generalization ability
Multi-Scale Training	Enabled	Randomized input resolution
Loss Function	RT-DETR Detection Loss	Combined classification, confidence, and bounding box regression loss
Evaluation Metrics	Precision, Recall, mAP@0.5, mAP@0.5:0.95	Multi-level detection quality

## 5. Experiment Result

### 5.1. Comparative experimental results

#### 5.1.1. NEU-DET.

To comprehensively validate the effectiveness of the proposed method in industrial surface defect detection, systematic comparisons are conducted on the NEU-DET dataset against several mainstream detection models. The compared methods include lightweight YOLO series networks such as YOLOv5, YOLOv6, YOLOv7, and YOLOv8, as well as high-complexity Transformer-based architectures like DETR. The recently proposed optimized lightweight model BMA-YOLO is also included. All models are evaluated under the same training strategy and performance metrics to ensure fairness and reliability. The experimental results are shown in Table 2.

**Table 2:** Comparison of Defect Detection Results on NEU-DET Dataset.

Methods	P/%	R/%	F1 score	mAP@0.5/%	mAP@0.5:0.95/%	FPS	Params(M)
YOLOv5 [45]	71.2	72.5	0.72	74.5	39.2	248.1	25.1
YOLOv6 [46]	69.8	71.1	0.70	75.1	40.0	175.0	34.9
YOLOv7 [47]	70.5	73.3	0.72	75.7	40.7	146.2	36.9
YOLOv8 [48]	68.9	70.6	0.69	73.6	38.9	170.6	25.9
DETR [49]	76.0	64.9	0.69	70.3	35.9	154.7	26.55
IF-YOLO [50]	72.3	69.1	0.71	74.8	39.7	170.7	27.31
PA-YOLO [51]	73.5	70.8	0.72	75.4	40.2	183.0	31.6
YOLO-SRSA [52]	74.1	71.5	0.73	76.2	41.0	88.4	67.3
LEAD-Net [53]	75.0	72.1	0.74	77.1	42.0	105.7	45.8
SMF-DETR [54]	74.6	70.9	0.72	75.8	41.3	97.3	42.50
BMA-YOLO [55]	75.5	71.2	0.74	77.7	43.1	163.2	36.8
Liu et al. [56]	–	–	–	**81.6**	–	46.1	11.65
**Ours**	**76.4**	72.8	**0.75**	**78.9**	**44.3**	103.5	43.21

The experimental results on the NEU-DET dataset indicate that the proposed method achieves competitive overall performance compared with mainstream object detection models, particularly showing advantages in F1 score and mAP@0.5:0.95. It should be noted that the result of Liu et al. [56] is directly cited from the experimental settings and reported results in their original paper, and is therefore included here as a reference comparison. Although the method does not outperform all compared approaches in mAP@0.5 and parameter efficiency, the results still demonstrate the effectiveness of the semantic-guided query enhancement mechanism and the hierarchical attention fusion structure. These designs improve semantic representation and cross-scale feature interaction, thereby enhancing the model’s robustness and detection accuracy for defect regions with complex textures and varying scales.

#### 5.1.2. DAGM2007.

To further validate the applicability and generalization capability of the proposed method for industrial defect detection tasks, the DAGM2007 dataset with typical texture defect characteristics is selected as the second evaluation platform. This dataset includes various types of complex textures and background interference, posing higher demands on fine-grained perception and feature representation. In the experiments, the proposed method is compared with several mainstream detection frameworks, covering representative models with different structural types and parameter scales. The experimental results are shown in Table 3.

**Table 3:** Comparison of Defect Detection Results on DAGM2007 Dataset.

Methods	P/%	R/%	F1 score	mAP@0.5/%	mAP@0.5:0.95/%	FPS	Params(M)
YOLOv5	76.2	74.3	0.75	78.6	43.6	248.1	25.1
YOLOv6	75.0	73.5	0.74	78.9	44.0	175.0	34.9
YOLOv7	77.1	75.3	0.76	80.3	45.5	146.2	36.9
YOLOv8	74.8	73.2	0.74	77.8	42.7	170.6	25.9
DETR	77.3	70.2	0.73	75.0	40.2	154.7	26.55
IF-YOLO	76.5	73.9	0.75	79.4	44.3	170.7	27.31
PA-YOLO	77.0	74.1	0.75	80.0	45.0	183.0	31.6
YOLO-SRSA	77.6	74.6	0.76	81.2	46.0	88.4	67.3
LEAD-Net	77.8	75.0	0.76	81.6	46.3	105.7	45.8
SMF-DETR	77.4	74.2	0.75	80.5	45.4	97.3	42.50
BMA-YOLO	78.0	74.8	0.76	82.1	46.9	163.2	36.8
**Ours**	**79.1**	**76.0**	**0.77**	**84.7**	**48.1**	103.5	43.21

The experimental results show that the proposed method achieves better overall performance than existing mainstream detection models on the DAGM2007 dataset, with notable improvements in both F1 score and mAP. This result is attributed to the semantic-guided query enhancement mechanism, which effectively strengthens the model’s semantic representation of defect targets. In addition, the hierarchical attention fusion structure improves the precision and discrimination of target localization under multi-scale background conditions. Together, these components help achieve a good balance between high accuracy and strong generalization capability.

#### 5.1.3. PCB-DET.

To further evaluate the adaptability and generalization performance of the proposed method in high-precision defect detection tasks, comparative experiments are conducted on the representative industrial dataset PCB-DET. This dataset includes various types of complex circuit board defects and places higher demands on detection accuracy and semantic modeling capability. The experiments involve comparisons with the YOLO series, Transformer-based architectures, and the advanced BMA-YOLO model, covering different paradigms of lightweight design and high-performance modeling. The experimental results are shown in Table 4.

**Table 4:** Comparison of Defect Detection Results on PCB-DET Dataset.

Methods	P/%	R/%	F1 score	mAP@0.5/%	mAP@0.5:0.95/%	FPS	Params(M)
YOLOv5	80.6	77.3	0.79	83.5	47.8	248.1	25.1
YOLOv6	79.2	76.0	0.77	84.1	48.5	175.0	34.9
YOLOv7	81.0	78.6	0.80	84.8	49.1	146.2	36.9
YOLOv8	77.9	75.8	0.77	82.4	46.9	170.6	25.9
DETR	83.3	72.1	0.77	78.9	43.2	154.7	26.55
IF-YOLO	81.8	77.0	0.79	84.7	48.9	170.7	27.31
PA-YOLO	82.4	77.5	0.79	85.3	49.4	183.0	31.6
YOLO-SRSA	82.9	78.1	0.80	85.9	50.1	88.4	67.3
LEAD-Net	83.5	78.4	0.80	86.0	50.4	105.7	45.8
SMF-DETR	82.7	77.2	0.79	85.1	49.0	97.3	42.50
BMA-YOLO	84.0	77.8	0.80	86.3	51.0	163.2	36.8
**Ours**	**85.2**	**79.1**	**0.82**	**87.4**	**52.3**	103.5	43.21

Table 4 presents the comparative results on the PCB-DET dataset. It can be observed that the proposed method achieves relatively strong overall performance among the compared detectors, reaching 85.2% in Precision, 79.1% in Recall, 0.82 in F1 score, 87.4% in mAP@0.5, and 52.3% in mAP@0.5:0.95. Compared with representative YOLO-based methods, DETR-based variants, and several recent improved models, the proposed approach shows a certain advantage in balancing detection accuracy and localization quality, especially under the stricter mAP@0.5:0.95 metric, which suggests that the model may provide more stable bounding box regression and finer defect region fitting on PCB images with complex structures and small-scale targets. At the same time, although the proposed method does not achieve the highest FPS and its parameter scale is moderately larger than that of some lightweight baselines, the overall results indicate that the introduced modules contribute positively to improving comprehensive detection performance while maintaining a practical inference efficiency.

### 5.2. The impact and analysis of the number of layers of the hierarchical attention module on experimental results

To further investigate the role of model architecture in industrial defect detection, this section explores the influence of the depth of the hierarchical attention module on the network’s overall representation capability. By progressively adjusting the number of attention layers, we aim to examine how the structural depth affects the model’s ability to capture cross-scale semantic dependencies and refine spatial feature interactions. This analysis provides insights into the trade-off between modeling capacity and computational complexity, and helps guide the optimal configuration of the attention mechanism for high-precision industrial scenarios.

**Fig 7:**
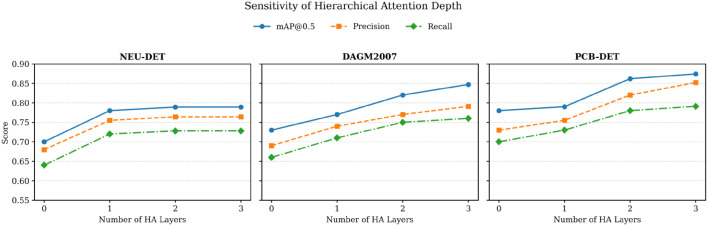
The impact of the number of layers of the hierarchical attention module on the experimental results.

As shown in Fig 7, increasing the number of layers in the hierarchical attention module consistently leads to improvements in mAP@0.5, precision, and recall across all three datasets (NEU-DET, DAGM2007, and PCB-DET). This indicates that a deeper attention structure enhances the model’s ability to aggregate and represent semantic features across different scales. Notably, on the PCB-DET dataset, the most significant performance gain is observed when the number of attention layers reaches three, suggesting that deeper attention mechanisms are particularly effective in modeling key regions under complex structures and sparsely distributed defects.

On the other hand, the magnitude of improvement varies among different evaluation metrics. The increase in mAP@0.5 is the most stable, followed by precision, while recall exhibits a comparatively slower improvement. This suggests that deeper attention layers are more beneficial for improving localization accuracy and confidence, whereas the recall of extremely rare targets may face diminishing returns. Therefore, in practical deployment scenarios, it is advisable to balance performance gains with computational overhead by selecting an optimal depth configuration based on specific task requirements.

The observed saturation and diminishing returns with deeper HAF layers mainly come from three factors. First, the cross-scale attention already achieves sufficient semantic alignment after a small number of layers, and subsequent layers tend to refine similar correspondence patterns, producing smaller marginal gains in feature discrimination. Second, deeper attention stacks progressively increase inter-token mixing, which may smooth fine-grained defect cues and reduce the distinctiveness of extremely small or low-contrast targets, thereby limiting recall improvements. Third, additional layers increase optimization difficulty and may introduce overfitting to dataset-specific texture statistics, especially when the number of rare defect instances is limited, which constrains further gains in precision and recall. These effects jointly lead to a performance plateau beyond the optimal depth.

### 5.3. Ablation experiment results

To comprehensively verify the actual contribution of each module to performance improvement, we design a series of ablation experiments. These experiments systematically evaluate the independent effects and collaborative interactions of key structures. By gradually removing or replacing specific components, we can effectively reveal how each module influences feature extraction, semantic representation, and detection accuracy. Such experiments help clarify the performance composition of the model and provide theoretical support for further structural optimization and practical deployment.

The ablation results in Table 5 show that both QEM and HAF consistently improve detection performance across NEU-DET, DAGM2007, and PCB-DET. Introducing QEM mainly enhances semantic-guided query discrimination, leading to stable gains in recall and F1 score, while introducing HAF strengthens cross-scale feature aggregation and semantic consistency, yielding consistent improvements in mAP@0.5 and mAP@0.5:0.95. In addition, removing ${ℒ}_{unc}$ from the full configuration causes a consistent performance drop on all datasets, for example, F1 decreases from 0.75 to 0.73 on NEU-DET, from 0.77 to 0.75 on DAGM2007, and from 0.82 to 0.79 on PCB-DET, indicating that uncertainty consistency regularization contributes to more stable cross-branch predictions.

**Table 5:** Ablation study of the proposed modules on three datasets.

Dataset	Model	P/%	R/%	F1 score	mAP@0.5/%	mAP@0.5:0.95/%
NEU-DET	Baseline	66.4	66.1	0.66	72.6	41.2
	+ QEM	69.9	72.6	0.71	75.8	42.3
	+ HAF	71.0	70.3	0.72	76.1	43.0
	+ ALL	**76.4**	**72.8**	**0.75**	**78.9**	**44.3**
	+ ALL w/o ${ℒ}_{unc}$	73.8	71.5	0.73	77.3	43.5
DAGM2007	Baseline	70.2	68.5	0.69	81.7	45.6
	+ QEM	73.8	71.2	0.72	84.5	47.3
	+ HAF	74.1	70.7	0.72	84.2	47.9
	+ ALL	**79.1**	**76.0**	**0.77**	**84.7**	**48.1**
	+ ALL w/o ${ℒ}_{unc}$	76.4	73.1	0.75	84.0	47.6
PCB-DET	Baseline	74.1	70.3	0.72	79.4	44.7
	+ QEM	76.3	72.1	0.74	81.8	46.0
	+ HAF	75.5	73.0	0.74	82.2	46.5
	+ ALL	**85.2**	**79.1**	**0.82**	**87.4**	**52.3**
	+ ALL w/o ${ℒ}_{unc}$	82.7	76.2	0.79	85.9	50.4

When QEM and HAF are combined, the model achieves the best results on all three datasets, indicating strong complementarity between semantic-guided query enhancement and hierarchical attention fusion. The combined configuration provides more accurate multi-scale localization and more robust defect feature representation under complex backgrounds, resulting in the most comprehensive improvements across precision, recall, F1 score, and mAP metrics. Moreover, the comparison between +ALL and +ALL w/o ${ℒ}_{unc}$ further confirms that ${ℒ}_{unc}$ improves both localization quality and semantic consistency, as reflected by the simultaneous reductions in mAP@0.5 and mAP@0.5:0.95 when it is removed.

### 5.4. Qualitative results

#### 5.4.1. NEU-DET.

**Fig 8:**
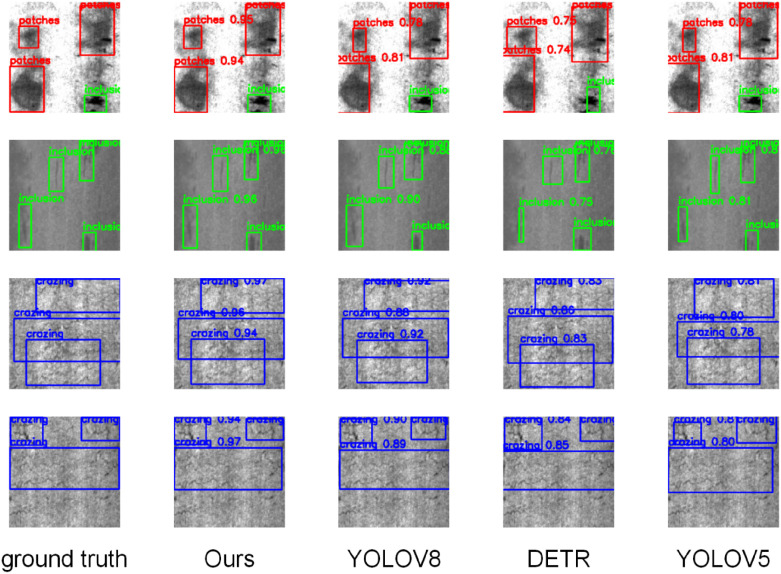
Qualitative experimental results on the NEU-DET dataset.

This paper first presents the qualitative experimental results of the NEU-DET dataset, as shown in Fig 8

As shown in the qualitative results of Fig 8, the proposed model demonstrates superior defect localization accuracy and boundary fitting capability on the NEU-DET dataset. Compared with mainstream methods such as YOLOv8, DETR, and YOLOv5, our approach achieves more precise identification of fine-grained defects like “patches” and “crazing” in densely populated regions, along with higher confidence scores and clearer bounding box alignment. Notably, under complex texture backgrounds, YOLOv8 and DETR suffer from missed detections and ambiguous overlapping boxes, whereas our method maintains consistent boundary delineation and accurate category recognition. These results indicate that the introduced semantic guidance and hierarchical attention mechanism effectively enhance the model’s fine-grained representation and semantic perception capabilities.

#### 5.4.2. DAGM2007.

**Fig 9:**
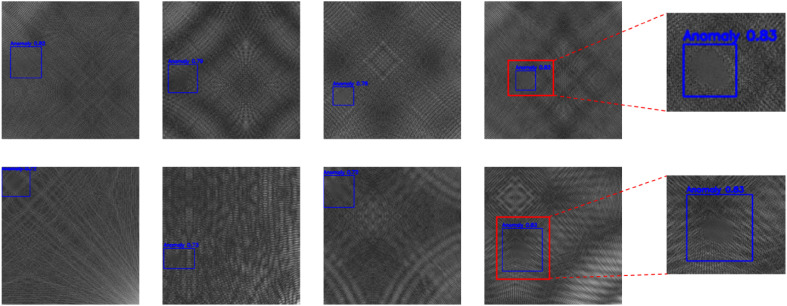
Qualitative experimental results on the DAMG2007 dataset.

This paper further presents experimental results on the DAGM2007 dataset. For a more intuitive presentation, this dataset only provides the qualitative results of the algorithm used in this paper, as shown in Fig 9.

As illustrated in the qualitative results on the DAGM2007 dataset in Fig 9, the proposed method exhibits strong performance in locating and identifying subtle defects under complex texture backgrounds. Across multiple representative samples, no instances of missed detection, positional deviation, or low confidence scores are observed. The proposed model accurately delineates defect boundaries, with detection boxes more tightly aligned to the target regions and higher confidence levels. For instance, the two enlarged results on the right clearly show that the model can precisely capture anomalous areas even under low-contrast textures, demonstrating enhanced robustness and perception capability in the presence of background interference. These improvements can be attributed to the introduction of the hierarchical attention structure and the semantic enhancement mechanism.

#### 5.4.3. PCB-DET.

**Fig 10:**
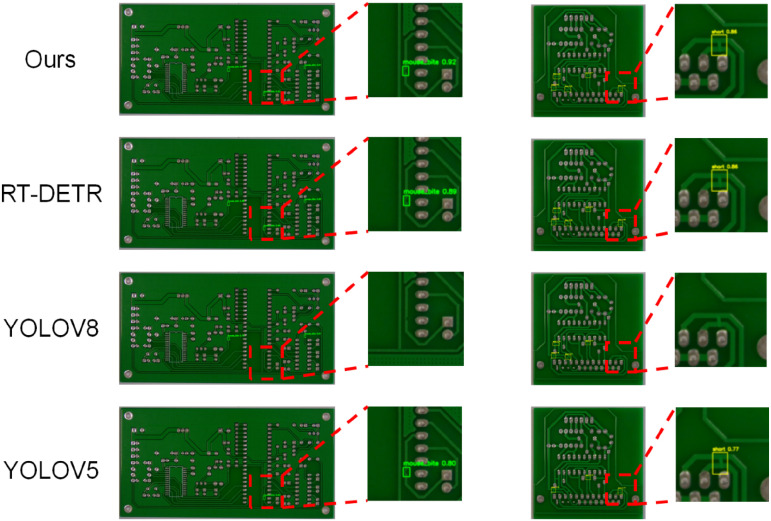
Qualitative experimental results on the PCB-DET dataset.

Finally, qualitative results on the PCB-DET dataset are given, and the experimental results are shown in Fig 10.

Fig 10 presents the qualitative detection comparison results on the PCB-DET dataset. It can be observed that the proposed method demonstrates superior perceptual ability and boundary fitting accuracy, particularly in regions containing subtle defects and densely structured components. In the enlarged detail views on the right side of the figure, our model accurately localizes challenging defects such as “blur” and “missing copper,” and produces clear and complete bounding boxes. In contrast, YOLOv5 and YOLOv8 suffer from missed detections or positional deviations, while RT-DETR, despite partially hitting the target areas, exhibits imprecise boundary delineation with vague enclosures.

Further observations reveal that the proposed method maintains stable detection performance across varying object densities and shapes, with bounding boxes closely overlapping the actual defect regions. This indicates that the introduced semantic guidance and hierarchical attention mechanism effectively enhance the model’s contextual modeling capability and cross-scale consistency. In comparison, other baseline methods tend to produce false detections or low confidence scores when dealing with small-scale anomalies or complex backgrounds, validating the superior generalization ability and practical applicability of our method in industrial scenarios characterized by sparse and structurally heterogeneous defects.

### 5.5. Grad-Cam experimental results and correlation analysis

**Fig 11:**
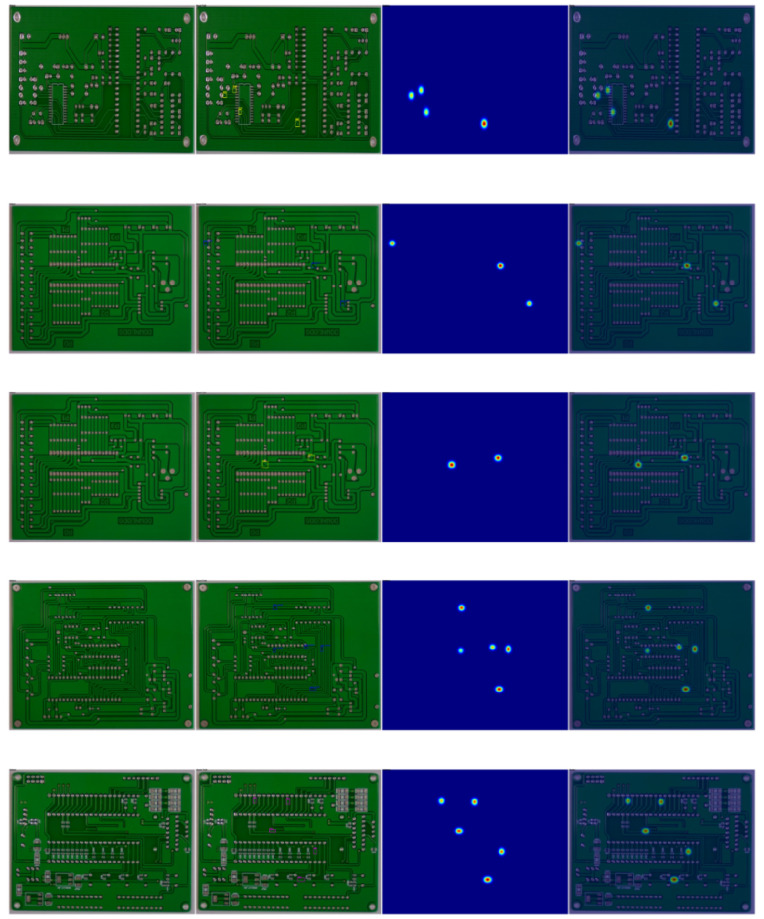
Grad-Cam experimental results on the PCB-DET dataset.

This paper first presents the Grad-Cam experimental results of PCB-DET, which are shown in Fig 11.

As shown in the Grad-CAM visualization results, the proposed model accurately focuses its attention on defect regions within the PCB-DET dataset, significantly highlighting the response intensity of critical areas. In each group of images—from the original image to the heatmap and the overlay—the model exhibits strong localization capability for subtle anomalies while effectively suppressing false activations in background regions. This precise attention response not only enhances the model’s discriminative ability toward target defects but also verifies the effectiveness of the semantic guidance mechanism and hierarchical attention structure in directing the model to focus on key areas, thereby laying a solid foundation for subsequent accurate detection and decision-making.

**Fig 12:**
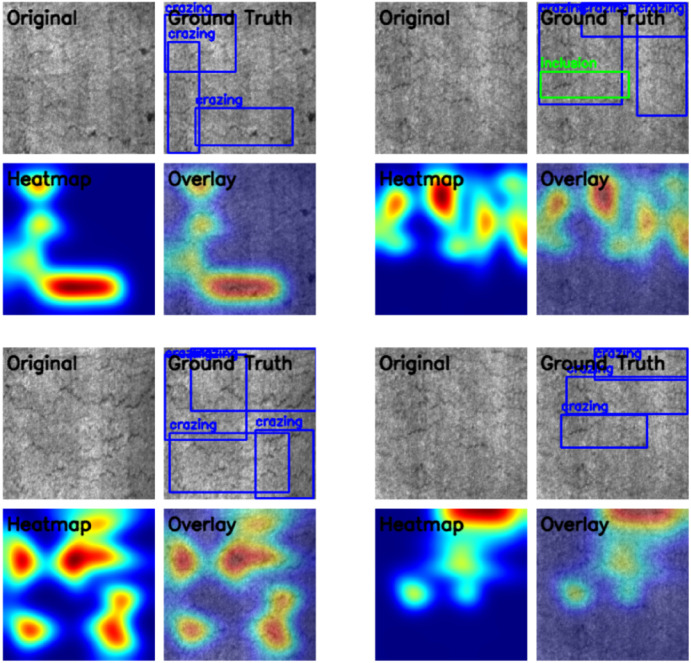
Grad-Cam experimental results on the NEU-GRAD dataset.

Finally, this paper also presents the experimental results of Grad-Cam on the NEU-DET dataset,‌‌ as shown in Fig 12.

### 5.6. Reliability analysis under noisy labels

**Fig 13:**
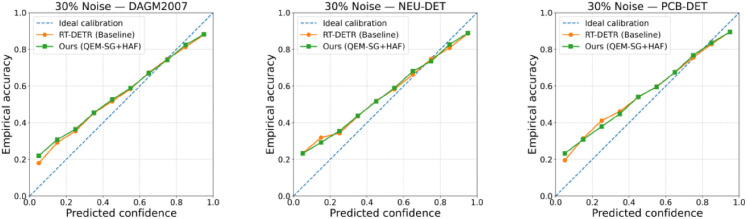
Reliability diagrams at 30% label noise on NEU-DET, DAGM2007, and PCB-DET. Compared with the RT-DETR baseline, our QEM-SG + HAF method yields confidence–accuracy curves closer to the ideal diagonal, indicating better calibration under noisy annotations.

In real-world industrial inspection tasks, models must not only maintain stable overall accuracy but also ensure that their prediction confidence truly reflects the probability of a sample being correctly classified—that is, they must be well calibrated. In particular, in the presence of label noise, models are prone to overconfidence or underestimation of uncertainty, which weakens the interpretability and reliability of detection results. To this end, we conducted reliability analysis on the RT-DETR baseline model and the proposed QEM-SG + HAF method under 30% label noise conditions. The relationship between prediction confidence and empirical accuracy is plotted, as shown in Fig 13.

The results show that, under 30% label noise, the confidence-accuracy curves of the RT-DETR baseline model on all three datasets deviate to varying degrees from the ideal diagonal, indicating overconfidence in noisy environments. However, the overall curves of the proposed QEM-SG + HAF method are closer to the ideal diagonal, with particularly significant improvements on the NEU-DET and PCB-DET datasets. This demonstrates that this method can better maintain consistency between prediction confidence and true accuracy in noisy labeling scenarios.

Further comparisons across different datasets reveal that noise has a more significant impact on the calibration of NEU-DET and DAGM2007, while the overall trend for PCB-DET remains relatively stable. This demonstrates that despite the varying characteristics of the datasets, our method improves model reliability in all three scenarios. These experimental results not only validate the proposed module’s superior accuracy but also demonstrate its robustness and practical value in terms of calibration under noisy labeling conditions.

## 6. Discussion

Despite the consistent gains brought by QEM, HAF, and the uncertainty regularization, the proposed approach remains a fully supervised detection framework and therefore inherits several intrinsic limitations. From a theoretical perspective, the optimization objective is dominated by the empirical risk on the annotated training distribution, which makes the learned decision boundary sensitive to extreme class imbalance and long-tail defect frequencies; as a result, the model may bias toward frequent defect patterns and exhibit degraded recall for rare categories even when overall mAP is high. Moreover, for unseen defect types or distribution shifts in texture, illumination, and manufacturing processes, supervision-driven feature alignment may not guarantee semantic invariance, and the learned representations can suffer from domain-specific overfitting, leading to uncertain generalization when novel defects appear. Future work will investigate more robust learning paradigms that reduce annotation dependence and improve open-set generalization, including imbalance-aware optimization, uncertainty-calibrated training, and self-supervised or weakly supervised adaptation strategies to better handle rare defects and unseen defect categories in real industrial deployments.

## 7 Conclusion

This paper addresses key challenges in industrial surface defect detection, including weak structural modeling, insufficient semantic representation, and poor scale adaptability. A high-performance detection model based on the RT-DETR architecture is proposed, incorporating a Semantic-Guided Query Enhancement Mechanism and a Hierarchical Attention Fusion Structure. These components jointly optimize the detection pipeline across multiple dimensions, including feature generation, semantic guidance, and multi-scale interaction. Extensive experiments conducted on three representative industrial defect datasets—NEU-DET, DAGM2007, and PCB-DET—demonstrate the superiority of the proposed method over mainstream approaches. Specifically, the model achieves mAP@0.5 scores of 78.9%, 84.7%, and 87.4%, and mAP@0.5:0.95 scores of 44.3%, 48.1%, and 52.3%, respectively, validating its robustness and accuracy in detecting small objects under complex backgrounds. Furthermore, Grad-CAM-based visualization experiments confirm the model’s strong capability in target focusing and boundary awareness, highlighting its high practical value for industrial applications.

Looking ahead, model lightweighting, unsupervised learning capabilities, and cross-modal perception will become key research directions in industrial visual inspection. On one hand, with the widespread deployment of edge computing and smart manufacturing equipment, reducing model size and inference latency without sacrificing detection accuracy will be essential for real-world deployment. On the other hand, due to the difficulty of obtaining precise annotations for defect samples in practical scenarios, weakly-supervised or unsupervised defect detection methods will exhibit greater potential. In addition, integrating multimodal data sources such as thermal imaging, X-ray, and structured light is expected to overcome current perceptual limitations in detecting concealed defects and inspecting composite materials. In summary, the proposed architecture offers a novel pathway for structural optimization and performance enhancement in industrial defect detection, while also laying a solid foundation for multi-task integration and real-world deployment.
